# Using Virtual Technology for Fear of Medical Procedures: A Systematic Review of the Effectiveness of Virtual Reality-Based Interventions

**DOI:** 10.1093/abm/kaab016

**Published:** 2021-04-03

**Authors:** Ayşenur Kılıç, Ashley Brown, Işıl Aras, Rita Hui, Jennifer Hare, Lyndsay D Hughes, Lance M McCracken

**Affiliations:** 1 Health Psychology Section, Psychology Department, Institute of Psychiatry, Psychology and Neuroscience, King’s College London, Guy’s Hospital, London Bridge, London, UK; 2 School of Orthodontics, Jacksonville University, Jacksonville, FL, USA; 3 Dental Psychology Service, Guy’s and St Thomas’ NHS Trust, London, UK; 4 Psychology Department, Uppsala University, Uppsala, Sweden

**Keywords:** Virtual reality, Medical procedures, Pain, Anxiety

## Abstract

**Background:**

Innovations in virtual reality (VR) technologies have improved the adaptability of its use in therapeutic settings, and VR has shown to be a promising treatment for fear of medical procedures, with research increasing in this area in recent years.

**Purpose:**

This review aims to collate evidence for the impact of VR on fear of medical procedures.

**Methods:**

CENTRAL (Cochrane), MEDLINE, EMBASE, and PsychINFO databases were searched up to October 2020. A mix of experimental and case–control studies were included for review, which evaluated the effectiveness of VR for fear, anxiety, and pain of medical procedures for people with needle phobia, dental phobia, claustrophobia of medical scans, and burn wound care anxiety. Risk of bias (RoB) was assessed by Cochrane and ROBINS-I tools.

**Results:**

Twenty-eight studies were selected. Some studies included mixed participant groups of young people adults. The interventions varied, with VR used for distraction, hypnosis, or exposure. These were shown to be effective for reducing fear of medical procedures. However, effectiveness for blood-injection-injury phobias and burn wound care patients was unclear.

**Conclusions:**

Evidence on the effectiveness of VR suggests that it does decrease fear of medical procedures in some situations. However, the RoB assessment illustrated a poor quality of studies across those included in this review, limiting the ability to draw firm general conclusions from the study findings. There is a need for further research exploring the use of VR technologies in the management of anxiety in physical health care settings.

## Teaser

Usage of virtual reality as a form of distraction, hypnosis or exposure can reduce fear of medical procedures like dental phobia.

Recent innovations in computer graphic technologies have revolutionized the experience of virtual reality (VR) and generated interest in its application for addressing real-life problems. As a result of this innovation, VR has become an important topic for scientific researchers. Several psychological and neuropsychological studies have been conducted to explore the adaptability of VR into psychological and neuropsychological research [1]. This review will aim to investigate the usage of virtual reality therapies (VRTs) within one specific domain of this research, including fear of medical procedures.

## Description of Condition

Fear of medical procedures (which is also known as procedural anxiety or tomophobia) is characterized by experiencing extreme distress and anxiety whilst participating in medical procedures. The most common types of these fears are blood-injection-injury (BII) phobias, including dental phobia, as well as the medical procedure anxiety [2].

Fear of medical procedures is a significant problem for patients and health care professionals. Indeed, many patients experience such fear of medical, dental, or surgical procedures and refuse to participate in procedures or avoid appointments entirely as a result [3]. Procedural anxiety is frequently recognized in people who need to take part in medical procedures regularly (e.g., Byers et al. [4]). Due to their constant exposure to these procedures, patients may attend their treatments with a recurrent experience of significant anxiety, sometimes associated with previous negative experiences [5].

Research recommends several techniques for patients with a fear of medical procedures. These include distraction techniques, such as listening to music or using imagery, relaxation techniques, and exposure therapy with patients who experience this fear [6]. In addition to these techniques, there is a growing interest in the application of VRT.

## Description of Intervention

VR is a form of computer technology (including a head-mounted display devices with headphones, screens, and monitor-tracking devices) that recreates life-like settings in a digitalized world and provides an opportunity for people to actively interact with this new environment [1]. Studies of VR for fear of medical procedures, broadly speaking, use it in one of two ways: (a) as a form of distraction or (b) as a type of exposure method. The first type of intervention aims to connect individuals’ attention to a different environment, thereby decreasing possible thought processes regarding the medical procedure. This could also be achieved by providing hypnosis via VR. On the other hand, VR interventions for the use of exposure, often referred to as virtual reality exposure therapy, aim to gradually expose people to the feared stimulus so as to decrease their anxiety toward the medical procedure. Essentially, both approaches are meant to reduce fear and anxiety, the first by blocking contact with the provoking situation, and second by reducing the capacity of situations to generate these feelings.

## How the Intervention Might Help

### VRTs for Dental Phobia

Dental phobia, also known as dentophobia or odontophobia, is the persistent and unreasonable fear of dental objects and situations, which is estimated to affect 11% of the UK population whilst nearly half of the adult population expressed feeling moderate to high levels of anxiety from dental treatments [6–10] also suggest that dental phobia is the most prevalent subtype of specific phobia.

Individuals with dental phobia avoid common dental treatment procedures, which negatively impacts their oral and general health by reducing dental attendance and uptake of dental care [11]. Effective management of dental phobia is crucial to improving oral health and quality of life. While researchers focused on the usage of VR to distract the patients’ attention away from the dental procedures to reduce the fear [12,13], some researchers have recently started to work on using exposure therapy through VR [14].

### VTRs for BII Types of Phobias

It is estimated that approximately 3% of the population experience needle or injection phobia (also named trypanophobia). BII phobia is another specific phobia characterized by extreme anxiety in relation to treatments involving one or all of these elements [15]. Due to the intense fear experienced, individuals avoid seeking medical or dental care, following recommended treatment, or providing blood for needed blood tests [16]. Because these are necessary for health and can even be lifesaving, treating needle phobia to improve patients’ adherence to appropriate health behaviors and recommendations for medical treatments is crucial.

### VRTs for Claustrophobia in Magnetic Resonance Imaging Screening

It is estimated that approximately 3% of the population experience anxiety when in a confined space, such as small rooms, lifts, tunnels, or public toilets [17]. Claustrophobic individuals may also experience restriction and suffocation fear as well as some characteristics of panic disorder or agoraphobia [18]. Many magnetic resonance imaging (MRI) devices include entering a small tunnel-like chamber, which can cause phobic responses in claustrophobic individuals. Due to this fear, participants may experience significant fear and anxiety whilst undertaking a scan or they may refuse to complete their MRI screening, posing potential threats to diagnostic ability of the medical team [19]. Many patients with a fear of MRIs control their anxiety by taking sedatives or using distraction techniques, such as listening to music or trying relaxation exercises. VR provides another mechanism to help patients manage anxiety during MRI scans. In a case study conducted by Garcia-Palacios et al. [19], the use of VR to distract individuals was found to be more effective than listening to music as a distraction method. Furthermore, individual’s anxiety levels significantly decreased compared to no distraction and the music distraction.

It is known that the experience of pain is strongly affected by psychological factors [20, 21]. It is common for burn patients to feel anxious and fearful about their daily wound care (cleaning and removing of the dead tissue) as wound care is associated with significant pain. Therefore, many patients have found it beneficial to use distraction techniques during their daily wound care in order to focus less on the pain and reduce related discomfort and distress [22]. The interactive and immersive nature of VR is recognized to decrease conscious attention to the medical procedure, thereby reducing the experienced pain by burn patients [23]. Indeed, Hoffman et al. [24] demonstrated that burn patients who participated in a virtual game using VR reported a decline in their perceived pain compared to patients who played Nintendo (e.g., a 2D game) while staples were removed from their tissues.

## Why This Review is Important

As demonstrated through the aforementioned literature, fear of medical procedures may cause individuals to avoid medical procedures due to the experience of anxiety and distress. Preliminary empirical studies show that VRTs may provide an effective treatment method to help patients with these negative experiences. Previous systematic reviews and meta-analyses [25–28] also support this claim. As VR technologies and published studies for medical procedures using VR have dramatically increased in recent years, an update is needed. Therefore, this review aims to explore the use of VR techniques and their effectiveness for reducing or managing the fear of medical procedures. Due to significant heterogeneity expected among the included studies, a meta-analyses was not performed.

## Objectives

This review aims to summarize the effectiveness of VR in therapeutic settings for reducing the fear of medical procedures in the general population, specifically among: dental fear (dentophobia), fear of needle (trypanophobia), claustrophobia for magnetic resonance imagining (MRI), and burn wound care anxiety. These procedures were chosen due to representing the most common fears of medical procedures.

## Methods

### Protocol and Registration

This review is based on the guidelines set out by the Cochrane Handbook for Systematic Reviews of Interventions [29] and the Preferred Reporting Items for Systematic reviews and Meta-Analyses (PRISMA) statement [30]. This study is registered to the PROSPERO system (www.crd.york.ac.uk/prospero/; registration number CRD42019152327) and can be found online.

### Eligibility Criteria

#### Types of studies

This review collected evidence from experimental and case–control studies on fear of needles and claustrophobia related to MRIs. Only experimental designs were included for the studies on dental fear and burn wound care anxiety (see Supplementary Appendix A). Studies without a full-text report (e.g., only having a conference abstract) were excluded from the review.

#### Types of participants

This review included people who were reported to experience fear, anxiety, or pain during medical procedures. There were no age, gender, or country restrictions for the included studies. Some of the studies had heterogeneous age groups, which included children and young people (0–18 years old) and adults (over 18 years old) together. Thus, while interpreting the study findings, age groups were not separated.

#### Types of interventions

Studies that mentioned the use of VR or immersive VR were included in the review. VR or immersive VR used for distraction, hypnosis, or exposure to the distressing stimulus/situation was included in this review. There were no differentiations for the types of VR settings, such as playing a game or observing a virtual environment. The intervention needed to be assessed in one or more sessions in order to meet inclusion criteria. Studies with other type of technologies (such as computer games without any kind of VR goggles etc., or telehealth) and traditional therapies (e.g., cognitive behavioral therapy) without the use of VR were excluded. Furthermore, there were no restrictions for who delivered the interventions (e.g., researchers, nurses, psychologists, dentists, and/or others).

#### Types of comparators

All studies required a comparison to baseline outcome measures (e.g., anxiety), to demonstrate change pre-use/postuse of VR. Studies for fear of needles and claustrophobia for MRI did not require a control group; however, this was stipulated for case–control studies on dental fear and burn wound care, typically comparing against usual care for procedural anxiety management (e.g., distraction techniques, reassurance, and challenging negative thoughts, using imagery, using relaxation techniques, sedatives, and exposure therapy).

#### Types of outcomes

The main outcomes of this review were: experience of fear during a medical procedure, experience of anxiety during a medical procedure, and experience of pain during a medical procedure. These outcome measures were chosen due to being the most commonly reported outcome measures in studies from preliminary literature searches. Anxiety and fear were included as separate outcomes due to reporting approach of included studies (e.g., usage of different scales to measure anxiety and fear). Any study with at least one or more of these outcomes were included. The study outcomes were expected to be measured by validated questionnaires, such as Visual Analogue Scale [31] for anxiety. Studies using only interviews or qualitative assessments to measure the study outcomes were excluded from this review.

#### Setting

Included studies were conducted within settings that required individuals to receive a medical procedure.

#### Language

Included papers were restricted to those written in the English language or had full English translations; papers with English abstracts without full English texts were excluded.

### Search Methods for Identification of Studies

#### Electronic searches

This review searched Cochrane Central Register of Controlled Trials (Central) and OVID databases, which are MEDLINE, EMBASE, and PsycINFO. Medical Subheadings (MeSH terms) were used to determine proper search terms; after peer review, the search terms outlined in Supplementary Appendix B were used for the database search.

### Data Collection and Analysis

#### Selection of studies

All study selection procedures were conducted by two independent reviewers (A.K. and A.B.). The studies searched through CENTRAL, EMBASE, MEDLINE, and PsycINFO up to October 2020 and merged into the reference management software, EndNote X8 (Clarivate Analytics, 2017). After data collection, the duplicates were first detected and removed by EndNote before manual deduplication. After screening for duplicates, eligibility criteria were assessed by title and abstract. The articles that did not include VR or target medical procedures were rejected and were not included in the full-text screening. Only papers that were identified as eligible for the full-text assessment were assessed for selection, and suitable studies were included in the review.

#### Data extraction and management

A data extraction table, which was informed by Cochrane [29] and PRISMA guidelines [30], was used to extract information from the selected studies. The main characteristics of the studies were tabulated to summarize the data Table 1.

**Table 1.** Data extraction table for the included studies

Reference	Country	Study design	Sample	Measurement of outcome	Data analysis	Results	Cohen’s *d* effect sizes (95% CIs)	Limitations
Dental phobia								
Al-Halabi et al. [36]	Syria	A single-blind RCT three-groups randomization: -Group A: Basic BGTs -Group B: VR -Group C: Table device and wireless headphones	102 participants (60 boys and 41 girls) Mean age: 7.4 years Range: N/A	Fear: N/A Pain: FACES Anxiety: N/A	One-way ANOVA	Fear: N/A Pain: No significant difference between the groups (*p =* .54) Anxiety: N/A	Fear: N/A Pain: LOI Anxiety: N/A	- Lack of blinding for external investigator - Improper size of VR Box for children
Asl Aminabadi et al. [37]	Iran	A single-blind RCT two-groups randomization: -Group 1: Treatment w/out VR -Group 2: Treatment w/out VR	117 participants (63 boys and 54 girls) Mean age: 5.4 years Range: 4–6 years	Fear: N/A Pain: FACES Anxiety: MCDAS(f) SCARED	Paired and independent *t*-tests	Fear: N/A Pain: Significant decrease in pain perception (*p* < .001) Anxiety: Significant decrease in state anxiety (*p* < .001)	Fear: N/A Pain: −1.51 (−1.92; −1.10) Anxiety: −4.49 (−5.14; −3.78)	- Possibility of carryover bias - Differences in baseline anxiety in each session due to external factors
Furman et al. [38]	USA	A within patient/split-mouth design three conditions: -No distraction -Watching movie -VR	38 participants (17 men and 21 women) Mean age: 45.9 years Range: N/A	Fear: N/A Pain: VAS Anxiety: N/A	Paired *t-*test	Fear: N/A Pain: Significantly lower during VR compared to the movie (*p* < .001) and control (*p* < .001). Anxiety: N/A	Fear: N/A Pain: −1.23_VRvsControl_ (−1.70; −0.73) −0.50_VRvsMovie_ (−0.95; −0.04) −0.71_MovievsControl_ (−0.24; −0.66) Anxiety: N/A	- Limited selection option of movie or VR environment - Simulator sickness (13% of participants) - Small sample size
Gujjar et al. [39]	Malaysia	A two-arm, parallel group RCT two-groups randomization: -VRET -IP	30 participants (12 males and 18 females) Mean age: 24.15 years Range: N/A	Fear: N/A Pain: N/A Anxiety: VAS MDAS DFS	A repeated-measures MANOVA	Fear: N/A Pain: N/A Anxiety: Significant difference in state and dental anxiety scores between the conditions at T2, T3, T4, T5 with large effect sizes favoring the VRET	Fear: N/A Pain: N/A Anxiety: 0.78 (0.02; 1.50)	- Lack of blinding of therapist and participants to the intervention - Inability to generalize the findings - Missing active control - Limited number of dental scenarios covered
Lahti et al. [40]	Finland	A randomized controlled single-center trial two-groups randomization: -Virtual reality relaxation (VRR) -TAU	255 participants (84 males and 171 females) Mean age: 52.5 years old Range: N/A	Fear: N/A Pain: N/A Anxiety: MDAS	Mixed-effects linear regression	Fear: N/A Pain: N/A Anxiety: Total and anticipatory dental anxiety decreased more in the VRR group than the TAU group for MDAS total score (*β* = –0.75*, p* < .001) and for anticipatory anxiety score (*β* = –0.43, *p* < .001).	Fear: N/A Pain: N/A Anxiety: −0.24 (−0.49; 0.002)	- Bias in age distribution as due to recruitment bias - Lower percentage of males in the sample
Niharika et al. [41]	India	A single-blind-controlled crossover RCT two-groups randomization: -VR -No VR	40 participants (22 boys and 18 girls) Mean age: 7.23 years Range: 4–8 years	Fear: N/A Pain: FACES Anxiety: MCDAS(f)	Paired and independent *t-*tests	Fear: N/A Pain: Significant decrease in pain perception (*p* < .001) Anxiety: Significant decrease in state anxiety (*p* < .001)	Fear: N/A Pain: −1.22 (−1.90; −0.49) Anxiety: −1.32 (−2.01; −0.57)	- Lack of blinding
Nunna et al. [42]	India	A single-blind-parallel RCT two-groups randomization: -VR -Counter stimulation	70 participants Mean age: N/A Range: 7–11 years	Fear: N/A Pain: FACES VAS VCARS Anxiety: Pulse rate	Student’s *t-*tests Repeated-measures ANOVA	Fear: N/A Pain: Similar decreases in the pain perception to local anesthesia needle prick in VR and counter-stimulation group Anxiety: Significant differences in pulse rates favoring the VR group (*p* < .05)	Fear: N/A Pain: −0.06 (−0.53; 0.41) Anxiety: −0.36 (−0.83; 0.12)	- Small sample size
Sweta et al. [43]	India	An RCT two-groups randomization: -VR -No VR	50 participants Mean Age: 39.72 Range: N/A	Fear: N/A Pain: VAS Anxiety: Pulse rate	*t-*tests	Fear: N/A Pain: VR group had lower scores than control group (*p* < .01) Anxiety: Pulse rate was lower in the VR group (*p* < 0.020)	Fear: N/A Pain: −1.13 (−1.71; −0.52) Anxiety: −0.68 (−1.23; −0.12)	- Small sample size - Passive VR environment - Participants’ blood pressure levels were not measured
Tanja-Dijsktra et al. [44]	UK	An RCT three-groups randomization: -Active VR -Passive VR -No VR	69 participants (28 males and 41 females) Mean age: 33.1 years Range: N/A	Fear: N/A Pain: N/A Anxiety: MDAS	ANOVA	Fear: N/A Pain: N/A Anxiety: A significant interaction was found between dental anxiety and VR (*p* = .023) for vividness of memories	Fear: N/A Pain: N/A Anxiety: 0.77	- Unable to comment on the temporal patterns in anxiety during VR due to not having real-time recordings of anxiety
Fear of needles								
Aydın et al. [45]	Turkey	A randomized controlled experimental study two-groups randomization: -VR (experimental group) -No intervention (control group)	120 participants (61 males and 59 females) Mean age: 10.4 years old Range: 9 to 12 years old	Fear: N/A Pain: FACES VAS Anxiety: N/A	Mann-Whitney U tests	Fear: N/A Pain: Significantly higher pain levels felt in the control group on WBFPS (*p* = .006,) and VAS (*p* = .039,) Anxiety: N/A	Fear: N/A Pain: −0.05 (−0.41; 0.30) Anxiety: N/A	- Lack of blinding - Conducted in a single center
Chad et al. [46]	USA	A pilot feasibility study	17 participants (11 males and 6 females) Mean age: Pediatric aged Range: N/A	Fear: MCFS Pain: FACES Anxiety: N/A	N/A	Fear: The average anticipated fear level after goggle use decreased with a near margin significance in 90% (*p* = .43) Pain: The use of VR headset provided 77% (*p* = .52) decrease in pain score. Anxiety: N/A	Fear: 0.42 (−0.60; 1.44) Pain: 0.34 (−0.68; 1.36) Anxiety: N/A	- Small sample size - Lack of control group
Dumoulin et al. [47]	Canada	An RCT three-groups randomization: -VR -Watching television -The Child Life program	59 participants (38 boys and 21 girls) Mean age: 13.37 years Range: 8–17 years	Fear: VAS Pain: VAS Anxiety: N/A	Repeated-measures ANOVAs	Fear: All three interventions were associated with a significant reduction in fear of pain (*p* < .05). Pain: All three interventions were associated with a significant reduction in pain intensity (*p* < .05). Anxiety: N/A	Fear: −0.52_VRvsTV_ (−0.11; 0.09) −0.70_VRvsTAU_ (−1.37; 0.01) −0.10_TVvsTAU_ (−0.74; 0.55) Pain: −0.49_VRvsTV_ (−1.08; 0.12) −0.16_VRvsTAU_ (−0.82; 0.52) 0.34_TVvsTAU_ (−0.32; 0.98) Anxiety: N/A	- Lack of blinding - Limitation of usage of VR an emergency room
Dunn et al. [48]	USA	An unblinded RCT two-groups randomization: -VR -Standard care distraction (SOC)	24 participants (20 males and 4 females) Median age: 13 years Range: 6–18 years	Fear: N/A Pain: VAS/FACES Anxiety: VAS/FACES	Kruskal–Wallis tests	Fear: N/A Pain: Both VR and SOC distraction techniques had a positive influence on procedural pain; no statistically significant differences were observed between Anxiety: The groups did not differ in procedural nervousness and worry (*p* = .67).	Fear: N/A Pain: LOI Anxiety: LOI	- Small sample size and single-institution design - Underpowered to evaluate the equivalence of procedure time and to compare VR versus SOC attributes
Gerçeker et al. [49]	Turkey	A parallel design RCT three-groups randomization: -VR: Rollercoaster -VR: Ocean Rift -No VR	136 participants (73 boys and 63 girls) Range: 5–12 years	Fear: CFS Pain: FACES Anxiety: CAM	Linear Regression Kruskal–Wallis	Fear: Fear point differences decreased by 4% and 6% respectively in the VR-Rollercoaster (*p* < .001) and the VR-Ocean Rift (*p* < .001), whilst control group increased by 20% (*p* < .001). Pain: Lower experience of pain for the VR groups in comparison to the control group (*p* < .001). Anxiety: Decreased by 5.4% for the VR-Rollercoaster (*p* < .001) and 12.6% for VR-Ocean Rift group (*p* < .001), whilst 34.1% increased for the control group (*p* < .001).	Fear: –1.45_VR-O vs Control_ (–1.90; –0.98) –1.73_VR-R vs Control_ (–2.20; –1.23) Pain: –0.99 _VR-O vs Control_ (–1.42; –0.55) –1.15 _VR-R vs Control_ (–1.58; –0.69) Anxiety: –1.65 _VR-O vs Control_ (–2.11; –1.16) –2.09 _VR-R vs Control_ (–2.59; –1.57)	- Lack of blinding in measurement process -Inclusion of limited age range - Limited range of VR environment choice
Gold and Mahrer [50]	USA	A parallel design RCT two-groups randomization: -Usual care -Usual care w/VR	143 participants (N/A) Mean age: 15.43 years Range: 10–21 years	Fear: N/A Pain: VAS CAS Anxiety: FAS CASI	Linear regression analyses	Fear: N/A Pain: The VR condition experienced significantly less procedural pain (*p* < .05). Anxiety: The VR condition experienced significantly less procedural anxiety and had better affect. Also, the VR group with high anxiety sensitivity experienced significantly less anxiety compared with standard care (*p* < .001)	Fear: N/A Pain: –0.32 (–0.65; 0.01) Anxiety: –0.27 (–0.60; 0.06)	- Lack of blinding
Jiang et al. [51]	Australia	A randomized parallel controlled pilot trial two-groups randomization: -VRET -Waiting list control group	43 participants (8 males and 35 females) Mean age: 23.44 years old Range: 18 to 48 years old	Fear: MFS MBPI Pain: N/A Anxiety: MDAS ADIS-5	Linear mixed model	Fear: Significant differences found in MFS Injections subscale between groups at 3 month follow-up (Hedge’s *g* = 0.63) Significant differences found in MBPI Injury subscale between groups at 1 week posttreatment (Hedge’s *g* = 0.64) and in MBPI Injection subscale at 3 month follow-up (Hedge’s *g* = 1.14) No significant group differences found Pain: N/A Anxiety: Small but no significant differences found in MDAS scores between groups at 3 month follow-up (Hedge’s *g* = 0.38).	Fear: –0.60 (–1.21; 0.01) Pain: N/A Anxiety: –0.27 (–0.87; 0.34)	- Small sample size - Possible placebo effect - Lack of blinding in group allocation process
Claustrophobia for MRI								
Garcia-Palacios et al. [19]	USA	A case report	25 years old and 49 years old Caucasian females	Fear: BAT Pain: N/A Anxiety: Rating on a 0–10 scale	N/A	Fear: VR distraction successfully reduced claustrophobic fear during the Mock MRI examination. However, music alone did not reduce a claustrophobic response. Pain: N/A Anxiety: N/A	Fear: N/A Pain: N/A Anxiety: N/A	- No statistical analyses carried out. - Small sample size
Wound care anxiety								
Chan et al. [52]	Taiwan	A crossover within subject design two-groups randomization: -VR -Usual care control	8 participants (7 boys and 1 girl) Mean age: 6.54 years Range: N/A	Fear: N/A Pain: FACES Anxiety: N/A	Paired *t*-tests One-way ANOVA	Fear: N/A Pain: Significant differences were found in both groups; no VR (*p* = .01) and VR (*p* = .04). Less pain seems to be experienced in the VR group during and after the dressing change though there is no statistical difference. Anxiety: N/A	Fear: N/A Pain: −1.42 (−2.40; −0.24) Anxiety: N/A	- Small sample size - Possibility of anticipated fear development before the study
Das et al. [53]	Australia	A within subject RCT	9 participants (6 boys and 3 girls) Mean age: 10 years Range: 5–18 years	Fear: N/A Pain: FACES Anxiety: N/A	Paired *t-*tests	Fear: N/A Pain: Statistically significant mean difference between the pharmacological analgesia only and VR coupled with pharmacological analgesia was 3.2 (*SD* 2.1, *p* < .001). Anxiety: N/A	Fear: N/A Pain: −1.16 (−1.97; −0.18) Anxiety: N/A	- Small sample size - Inability to generalize findings - Potential crossover bias due to repetitive testing
Faber et al. [54]	Netherlands	A within subject design	36 participants (N/A) Mean age: 27.7 years Range: 8–57 years	Fear: N/A Pain: VAT Anxiety: N/A	Paired *t-*tests	Fear: N/A Pain: Pain ratings during wound debridement were statistically lower when patients were in virtual reality on Days 1, 2, 3, and although not significant beyond Day 3. Anxiety: N/A	Fear: N/A Pain: 0.93 (0.24; 1.62) Anxiety: N/A	- Possibility of crossover bias
Hoffman et al. [55]	USA	Within-subject, within-wound care pilot RCT	48 participants (33 boys and 11 girls) Mean age: 12 years Age range: 6 – 17 years	Fear: N/A Pain: GRS PCS-C Anxiety: N/A	A paired *t-*test	Fear: N/A Pain: Significant reductions in worst pain during VR, pain unpleasantness, and in time spent thinking about pain during wound care (*p* < .05) Anxiety: N/A	Fear: N/A Pain: −1.30 (−1.74; −0.86) Anxiety: N/A	- Limited generalizability of the study to other populations
Khadra et al. [56]	Canada	One group, quasi-experimental pilot study	15 participants (6 girls and 9 boys) Mean age: 2.2 years Range: 2 months to 10 years	Fear: N/A Pain: FLACC Scale Anxiety: MSS PBCI	Freidman tests, Spearman’s rank correlation	Fear: N/A Pain: No statistically significant difference in observed pain, before, during, and after the procedure (*p* = .264). Anxiety: No statistically significant difference in procedural anxiety (*p* = .827)	Fear: N/A Pain: LOI Anxiety: LOI	- Small sample size - Lack of a control group
Khadra et al. [57]	Canada	A within-subject within-wound-care crossover design	38 participants (27 boys and 11 girls) Mean age: 21.9 months old or 1.8 years old Range: 6 months to 7 years old	Fear: N/A Pain: FLACC NRS-obs Anxiety: N/A	Wilcoxon signed-rank test	Fear: N/A Pain: Projector-Based Hybrid VR significantly reduced FLACC procedural pain levels (*p* = .026) Patients’ pain levels in NRS-obs were non-significant between both groups (*p* = .135) Anxiety: N/A	Fear: N/A Pain: LOI Anxiety: N/A	- Small sample size - Young age of participants does not allow for use of self-report assessments - Lack of blinding
Konstantatos et al. [58]	Australia	A prospective RCT 2-groups randomization: -VR Relaxation + IM -PCAI or IM alone	89 participants (N/A) Mean age: N/A Range: 18–80 years	Fear: N/A Pain: VAS Anxiety: BSAR	*t-*tests	Fear: N/A Pain: Pain intensity significantly differed with VR + PCA group mean of 3.7 and a PCA group mean of 2.3 (*p* = .031). Anxiety: Did not demonstrate any significant differences	Fear: N/A Pain: 0.47 (0.04; 0.90) Anxiety: LOI	- Providing only one session of psychological relaxation hypnosis may also result in distress to some participants
Maani et al. [59]	USA	A within-subject experimental design	12 male participants Mean age: 22 years Range: 20–27 years	Fear: N/A Pain: GRS Anxiety: N/A	A paired *t-*test	Fear: N/A Pain: Statistically significant differences in pain, time spent thinking about pain, and pain unpleasantness (*p* < .05) Anxiety: N/A	Fear: N/A Pain: −1.39 (−2.17; −0.41) Anxiety: N/A	- Lack of blinding - Small sample size
McSherry et al. [60]	USA	A within-subject, RCT study two-groups randomization: -IVR (and wound care procedure) -No IVR (only wound care procedure)	18 participants (13 male and 5 female) Mean age: 38.4 years old Range: N/A	Fear: N/A Pain: VNS Anxiety: VNS	Student’s *t*-test Chi-square analysis	Fear: N/A Pain: No significant difference in pain scores in IVR and No IVR group (*p* > .05) Total opioid administration for the dressing procedures in IVR group was significantly less than No IVR group (*p* = .02) Anxiety: No significant difference in anxiety scores in IVR and No IVR group (*p* > .05)	Fear: N/A Pain: 0.04 (−0.68; 0.75) Anxiety: 0 (−0.72; 0.72)	- Small sample size - 67% of participants had a history or opioid abuse, which may have affected preprocedure pain scores - using VNS to measure anxiety may not have been sensitive enough
Mott et al. [61]	Australia	A prospective RCT two-groups randomization: -VR -Basic cognitive therapy	In total, 42 participants (30 male and 12 female) Median age: 9 years Range: 3–14 years	Fear: N/A Pain: FLACC (3–4 year olds); FACES (4–8 year olds); VAS (8–14 year olds) Anxiety: N/A	*t*-tests Wilcoxon Rank-Sum Test ANOVA	Fear: N/A Pain: A significant decrease over time in the altered reality treatment group (*p* = .006) compared to control for the long dressing group (>30 min duration) Anxiety: N/A	Fear: N/A Pain: −3.46 (−4.34; −2.45) Anxiety: N/A	- A single software program may result in a limited appeal for older age groups -Heavy VR device
Van Twillert et al. [62]	Netherlands	A within-subject design three-groups randomization: -Standard care -VR -Self-chosen distraction method	19 participants (12 men and 7 women) Mean age: 30 years Range: 8–65 years	Fear: N/A Pain: VAT Anxiety: STAI	A two-way ANOVA	Fear: N/A Pain: VR distraction and watching television were the only distraction techniques that showed significant pain reductions (*p* < .05). The difference between VR and TV analgesia was not statistically significant. Anxiety: Reductions in anxiety scores were not significant.	Fear: N/A Pain: LOI Anxiety: 0.04 (−0.73; 0.80)	- Sample size - Lack of blinding

*ADIS-5* Anxiety and Related Disorders Interview Schedule for DSM-5—specific phobia module; *BAT* Behavioural Avoidance Test; *BGT* Behaviour Guidance Techniques; *BSAR* Burns Specific Anxiety Rating; *CAM* The Children’s Anxiety Meter; *CAS* Coloured Analogue Scale; *CASI* The Childhood’s Anxiety Sensitivity Test; *CFS* The Child Fear Scale; *CI* confidence intervals; *DFS* Dental Fear Survey; *FACES* The Wong-Baker FACES Pain Rating Scale; *FAS* Facial Affective States; *FCAI* FCA Infusion; *FLACC* Face, Legs, Activity, Cry, Consolability scale; *GRS* Graphic Rating Scale; *IM* intravenous morphine; *IP* information pamphlet; *LOI* lack of information; *MBPI* Multidimensional Blood Phobia Inventory; *MCDAS(f)* FACES version of the Modified Child Dental Anxiety Scale; *MCFS* McMurtry Children’s Fear Scale; *MDAS* Modified Dental Anxiety Scale; *MFS* Medical Fear Survey—Short Version; *MSS* The Modified Smith Scale; *N/A* not available; *NRS-obs* Pain-Numerical Rating Scale-observational; *PBCL* The Procedure Behaviour Check List Scale; *PCS-C* The Pain Catastrophising Scale for Children; *RCT* randomized controlled trial; *SCARED* Screen for Child Anxiety Related Disorders Questionnaire; *STAI* The State-version of the Spielberger State Anxiety Trait Anxiety Inventory; *TAU* treatment as usual; *VAS* visual analogue scale; *VAT* visual analogue thermometer; *VCARS* Venham’s Clinical Anxiety Rating Scale; *VNS* Verbal Numeric Scale; *VR* virtual reality; *VRET* virtual reality exposure therapy; *VR-O* VR-Ocean Rift; *VR-R* VR-Rollercoaster; *w/out* with or without.

#### Assessment of risk of bias in included studies

The possibility of having risk of bias (RoB) in the review was acknowledged and assessed using the “RoB” tool for randomized studies by the Cochrane Collaboration [29] and ROBINS-I for nonrandomized studies [32] (see Supplementary Appendix D).

#### Data synthesis

The studies were mainly grouped according to the relevant medical procedure (dental anxiety or pain, claustrophobia during MRI, burn wound care, and BII/needle phobia). Within these categories, the studies are summarized for their effectiveness on decreasing fear, anxiety, and pain in relation to the procedure. SWiM guidelines [33] were followed, and Cohen’s *d* effect sizes [34] and 95% confidence intervals were calculated for providing a narrative synthesis (see Table 1 and Supplementary Appendix E). A meta-analysis was not carried out due to the heterogeneous nature of the included studies in line with study protocol.

## Results

### Description of Studies

#### Results of the search

**Fig. 1.**
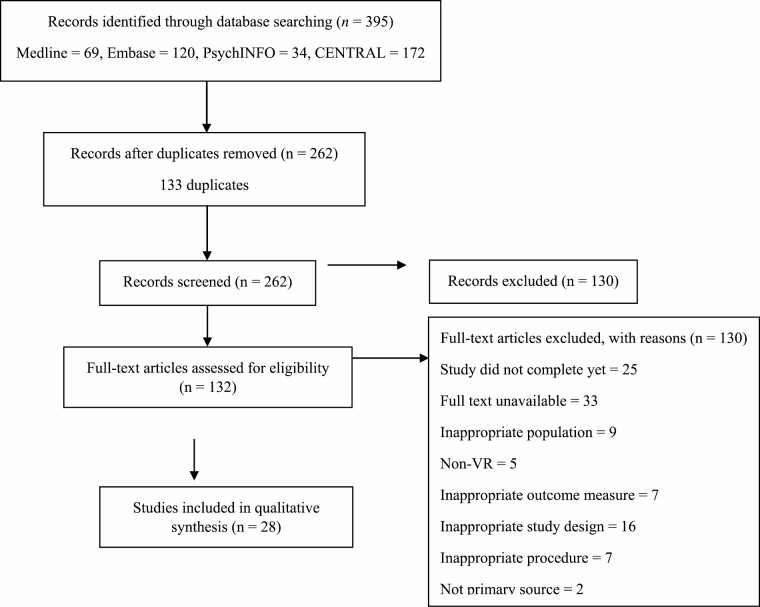
Preferred Reporting Items for Systematic reviews and Meta-Analyses statement flow diagram for the search results.

Figure 1 illustrates relevant studies identified through the database search performed independently by A.K. and A.B. Cohen’s Kappa agreement [35] between the independent reviewers was substantially high (0.85). In total, 28 studies included for qualitative synthesis.

#### Included studies

This systematic review only shared 25% or less of similarity for the included papers with previously mentioned reviews. Included studies were conducted in the following countries: the USA (*n* = 8), Australia (*n* = 4), Canada (*n* = 3), India (*n* = 3), Netherlands (*n* = 2), Turkey (*n* =2), Finland (*n* = 1), Iran (*n* =1), Malaysia (*n* =1), Syria (*n* =1), Taiwan (*n* =1), and the UK (*n* =1). Of the included studies, a total of 1,652 people participated. The mean sample size of these 28 studies was 59 participants (range 2–255 participants per study). No age restrictions were imposed within the inclusion/exclusion criteria; therefore, this review illustrated a heterogeneous age group, ranging between 2 months to 80 years old. The main outcomes of the included studies were anxiety (*n* = 15), fear (*n* = 5), and pain (*n* = 23). Furthermore, the guiding theoretical framework of the included studies was mostly based on distraction (*n* = 25), followed by hypnosis (*n* = 2) and exposure (*n* = 2).

The included studies used a wide variety of devices for creating a virtual world for the study participants (see Supplementary Appendix C). Studies involved various kinds of VR environments, including computer games (*n* = 14), natural environments (*n* = 5), cartoons (*n* = 4), adventure rides (*n* = 2), or outpatient clinics (*n* = 2).

#### RoB in included studies

The overall quality of included studies was low for randomized and nonrandomized studies (see Supplementary Appendix D for further information).

### Results for Each Condition

#### Dental phobia

Eight studies including 771 participants investigated the use of VR for distraction for dental phobia, whilst only one study used VR for exposure. Young people (*n* = 3) and adult (*n* = 5) samples were used in included studies and no differences were seen between them. The main study outcomes of these studies were pain (*n* = 6) and anxiety (*n* = 7; see Table 1). Studies used VR during dental procedures (e.g., periodontal scaling or root planning; *n* = 7) or to treat dental anxiety (*n* = 1).

All studies used the VR distraction method for the pain outcome. Nearly all these studies showed a significant decrease in experienced pain during VR, with only one study indicating no significant difference (*p* = .54). It seems that the use of VR as a distraction during a dental procedure may decrease experienced pain.

The outcome of anxiety was assessed by VR studies for both distraction and exposure, all of which demonstrated statistically significant decreases in the experience of anxiety for VR groups. Studies showed that VR used as a distraction, as well as for exposure, were both effective in decreasing the experienced anxiety relating a dental procedure.

#### The BII types of phobia

There were seven studies including 542 participants that investigated the use of VR for distraction (*n* = 6) or exposure (*n* = 1) for BII phobia. None of the studies reported the use of VR for hypnosis. Nearly all studies included child samples, whilst only one study included an adult group ranging between 18 and 48 and a mixed age group ranging between 10 and 21 years. The main study outcomes were pain (*n* = 6), fear (*n* = 4), and anxiety (*n* = 4). The VR distraction was used for various injection-related procedures (e.g., blood withdrawal or immunization).

Conflicting findings emerged. While some studies with a non-VR or usual care control (*n* = 4) reported statistically significant decreases for pain, studies with an active control (e.g., watching television, the Child Life program, and standard care distraction; *n* = 2) did not find any statistical differences between the groups. Similar trends were also seen in fear and anxiety outcomes. The use of VR as a distraction was an effective method to decrease the experience of pain, fear, and anxiety among participants for the fear of injection phobia. However, these improvements were no better than other active distraction techniques.

#### Claustrophobia in MRI screening

There was only one study [19] that investigated the use of VR for claustrophobia during an MRI scan. The main study outcomes were fear and anxiety using an anxiety rating scale and a behavioral avoidance test to measure outcomes. Two participants who were 25 and 49 years old were included as participants. VR or listening to music were used as distraction methods. Results illustrated that VR used as a distraction reduced the claustrophobic fear during the mock MRI, but music distraction alone did not.

#### Burn wound care

There were 11 studies with 334 participants that investigated the use of VR for distraction (*n* = 10) and hypnosis (*n* = 1) during the treatment of burn wound care. The use of VR for exposure was not applicable as a technique for the burn wound care population. Young people (*n* = 6), adult (*n* = 3), and mixed (*n* = 2) samples were used in the included studies. The main study outcomes were pain (*n* = 11) and anxiety (*n* = 4); fear was not separately reported in any of the studies. VR was used as a distraction method during the changes of dressing or the removal of dead skin tissue.

Nearly all studies (*n* = 9) reported that the VR distraction group demonstrated statistically significant decreases in the experience of pain. Only two studies indicated that VR did not result in any change on the experience of pain. Additionally, one study reported that, even though VR decreased the experience of pain, this decrease in pain was not significantly different from watching television as a form of distraction. Moreover, none of the studies reported statistically significant decreases in the experience of anxiety. Overall, the effectiveness of VR used for the application of distraction among burn wound care type of medical procedures consistently demonstrates improved pain outcomes, though results are mixed in its ability to reduce anxiety.

### Effectiveness of VR in Overall Study Outcomes and its Comparison to the Degrees Field of View of the VR Goggles

Standardized mean differences by using Cohen’s *d* [34] were calculated for posttest scores of fear, pain, and anxiety outcomes. Studies are grouped under four categories [34, 63]: small (0.2), medium (0.5), large (0.8), and very large (1.30).

Overall outcomes mostly demonstrated small effect sizes, followed by large, very large, and medium effect sizes. It is important to note that some of these small effect sizes were found in the comparison of control to distraction techniques (e.g., TV vs. treatment as usual; see Table 1 and Supplementary Appendix E). VR usage showed larger effect sizes for decreasing fear, pain, and anxiety outcomes in comparison to usual care or other distraction techniques.

No trend was found between the field of degree and effect sizes. However, careful consideration is needed for this finding as not all studies provide detailed information about the VR equipment used.

## Discussion

The main purpose of this systematic review was to investigate the current evidence on the effectiveness of VR-based interventions for reducing fear, pain, or anxiety of medical procedures. This review specifically focused on VR used for dental phobia, BII phobias, claustrophobia during MRI scans, and burn wound care. Twenty-eight research papers were selected based on the inclusion/exclusion criteria developed. Although the number of the studies was limited within each setting or condition, most of the studies showed significant reduction for the experience of fear, anxiety, or pain among participants. However, although the reduction in pain was clear for burn wound care studies comparing VR versus standard care, this reduction was not clear for the BII phobias and burn wound care when compared to active control.

The selected studies were heterogeneous with respect to patient groups, age, the study outcomes, and the study designs. Also, the RoB assessment exhibited significant risks across included studies in this review additionally limiting the ability to draw firm general conclusions.

Dental phobia has been associated with psychosocial impairment as a consequence of avoidance of dental care, resulting in deteriorating oral health [64, 65]. Berggren and Meynert [66] described the vicious cycle of dental anxiety: dental fear (often compounded by feelings of embarrassment) leads to the avoidance of dental care, which results in poor oral health, and poor oral health results in the need for more invasive (and typically more painful) dental care. Therefore, it is necessary to find a solution on how to best treat dental anxiety and phobia to prevent poor oral health in the future. As the literature points out, the complexity of the multifactorial etiology of dental anxiety, compensation for these factors seems to be a more efficient strategy to tackling the root cause (i.e., distraction works to increase better coping without necessarily overcoming the underlying fear). In this way, VR provides the tools to not only distract the patients’ attention from the procedure but also decreases the vividness of procedural memories especially in highly anxious people [44].

Clear negative cognitive impacts in the cycles of care can also be seen in procedural phobias (BBI phobias and claustrophobia during MRIs). Studies show that, after the hereditary factors, early traumatic experiences are thought to be the second highest reason for the acquisition of procedural phobias, which is also in line with conditioning theory [67, 68]. Similarly, this vicious cycle can be seen in the wound care: an early bad experience during a dressing change may lead individuals to experience increased pain and anxiety, which may then lead to the experience of increased anticipated anxiety [69] and higher pain perception. Increased anticipation of pain and anxiety may increase the chronic stress levels and this recurring theme would demonstrate itself in subsequent further pain and anxiety [70]. Upton [71] also shows that increased anxiety can decrease the tolerance and pain threshold, which would eventually make people more prone to experiencing greater pain. Therefore, VR may be an effective tool for its application to health care settings in order to decrease the chance of developing a vicious cycle of care and help decrease fear of medical procedures, ultimately increasing the quality of life.

Various methodologies, such as traditional distraction or exposure, have been used for the treatment of fear of medical procedures, and the effects of these methods, such as watching a movie, on pain, anxiety, and fear outcomes may be limited. In contrast, the use of VR from the earliest session may provide more immersive distraction possibly through emotional, cognitive and attentional processes (see Li et al. [72] for further discussion), which may lead to decreased pain and anxiety, along with the reduction of the chances of entering the negative cycle of medical phobias described above. Although the use of VR for the fear of medical procedures seems promising [73, 74], some studies show inconsistent results. Therefore, it is important for future research to invest in the application of VR to health care settings to better understand it’s potential role.

This systematic review followed the Cochrane and PRISMA guidelines for conducting a systematic review and published a protocol before conducting the systematic review, which increased the transparency of the study findings. Cohen’s Kappa agreement [35] between the independent reviewers indicated a high reliability for the inclusion of studies. However, this review provides only a narrative synthesis of the included studies and included case–control studies for some medical procedures (e.g., claustrophobia for MRI); therefore, some caution is needed when making interpretations or generalizations from these findings. Unfortunately, due to the limited research in the area and, therefore, the consequential heterogeneity of the included studies, meta-analyses could not be performed. Therefore, firm recommendations for clinical protocols are not possible at the present time.

VR-based interventions may reduce the anxiety and distress patients experience during medical procedures. Therefore, investigating its effectiveness is important for improving methods within the health care system. This systematic review revealed that VR-based interventions used distraction, hypnosis, or exposure, with the majority of studies utilizing VR for the purposes of distraction. Future research might usefully focus on its use for exposure and hypnosis techniques. Moreover, the most investigated medical procedure treated with VR was burn wound care. One may conclude that the use of VR for distraction as pain relief is more applicable to wound care over other procedures that are not necessarily associated with pain (e.g., MRI scans). Therefore, it is evident to say that there is a need for investigating other types of medical procedures, such as VR usage, for claustrophobia in MRI. Furthermore, VR research is closely affected by the technological innovations, which may increase patients’ involvement with the virtual world. In 2020, the VR market was forecasted to grow about 19 million dollars [75]. Accordingly, the worlds’ largest technology companies increased their shares spent on research and development expenditures of VR technologies [75]. Thus, it is expected that VR will become more immersive and cost-effective in the future due to this competitive mass marketing, which may increase analgesic effectiveness [76, 77]. Consequently, researchers need to follow technological innovations closely and implement these techniques in a clinical setting in order to use the most up-to-date and effective technologic innovations.

The use of VR technologies for the fear of medical procedures may influence clinical practice in future. If future studies support the findings of this systematic review, the application of VR systems to health care policies or within the NHS could improve patients’ satisfaction and decrease the amount of money spent on other traditional methods (such as cognitive behavioral therapy for reducing fear). Moreover, further application of VR technologies to the health care settings could improve adherence to medical procedures and health-related quality of life by allowing visits to a hospital or a health center to be a less fearful and more desirable experience.

In conclusion, the use of VR-based interventions appear effective to reduce the fear of medical procedures in some situations. However, the number of studies for different types of medical procedures were limited. Heterogeneity across studies was high, and methodological quality relatively low, meaning there is considerable RoB. Funding studies that can test the effectiveness of the new, more distracting VR systems in larger randomized controlled trials may lead to interventions that improve procedure acceptability, reduce negative patient outcomes (e.g., fear, anxiety, and pain), and reduce service costs. Thus, findings outlined in this review are clearly encouraging and point to future question to address.

## Supplementary Material

kaab016_suppl_Supplementary_Material
