# Supplementary material for: Using Virtual Technology for Fear of Medical Procedures: A Systematic Review of the Effectiveness of Virtual Reality-Based Interventions
Source: Ann Behav Med. 2021 Apr 3;55(11):1062–79. doi: 10.1093/abm/kaab016 (PMC8557375; doi:10.1093/abm/kaab016)
Supplement: kaab016_suppl_Supplementary_Material [file kaab016_suppl_supplementary_material.docx]

Appendix A

PICOS table for inclusion and exclusion criteria

|  | Inclusion criteria | Exclusion criteria |
| --- | --- | --- |
| Population | People with fear, anxiety, or pain for medical procedures; any age group (child [0-18 years old] and adult [over 18 years old]); any gender; population not restricted to the UK, will examine the papers from all over the world | People who do not express fear, anxiety, or pain towards a medical procedure |
| Interventions | Technology assigned interventions for psychological therapies; virtual reality therapy (VRT) for one or more session; for distraction, hypnosis, or exposure | Traditional and other forms of psychological therapies; other form of technology assigned interventions for psychological therapies such as telehealth and others |
| Comparator | Comparison of baseline with no-VR and after-VR change in the outcome measures (e.g., Anxiety). The studies with fear of needle, claustrophobia for MRI with/out a control group, whilst studies on dental phobia and burn wound care with a control group, such as usual care for procedural anxiety (e.g., distracting techniques, reassuring someone’s self and challenging negative thoughts, using imagery, using relaxation techniques, sedatives, exposure therapy). | Only virtual reality without any comparator |
| Outcomes | Fear; anxiety; pain of medical procedures (dental phobia, needle phobia, claustrophobia in MRI, wound care) | None |
| Study Design | Experimental and case-control study designs with a full text report for studies on fear of needle and claustrophobia for MRI, whilst only experimental study designs for the studies on dental phobia and burn wound care anxiety | Qualitative studies or cross-sectional studies for all types of procedural anxiety, and some case control studies without a full text report (e.g., conference abstract) |

Appendix B

Search terms for the relevant medical procedures

| Virtual reality, VR, immersive video, 360*video*, immersive reality therap*, virtual reality exposure therap*, VRET |
| --- |
| dentophobia, dental fear, dental anxiety, trypanophobia |
| fear of needle*, fear of injection*, needle phobia*, injection phobia*, blood phobia*, blood fear*, injury phobia*, injury fear*, blood-injection-injury phobia* |
| magnetic resonance imaging, MRI |
| claustrophobia |
| wound care, burn wound care, burn pain, wound pain |

Appendix C

Characteristics of VR in the included studies

| **Study** | **Guiding theoretical frameworks** | | | | **Virtual Reality Equipment** | **Virtual Reality Environment** |
| --- | --- | --- | --- | --- | --- | --- |
|  | **Distraction** | **Hypnosis** | **Exposure** |  | |  |
| **Dental Fear** |  |  |  |  | |  |
| Al-Halabi et al. (2018) | X |  |  | **Device:** The AV eyeglasses (BlackBug^TM^ Virtual Reality Glass 3D VR Box Headsets, China)  **Connected to:** a mobile phone (Asus Zenphone 2 DeluxeTM, ASUS R).  **Sound delivery:** a wireless headphone. | | A single episode of the cartoon series: “Tom and Jerry”, “Gumball”, “Pink Panter”, or “Sponge Bob” |
| Asl Aminabadi et al. (2012) | X |  |  | **Device:** i-glasses 920 HR Ilixco (920,000-pixel high resolution), Inc. Menlo Park, CA, USA | | A single episode of the cartoon series "Tom and Jerry" |
| Furman et al. (2009) | X |  |  | **Device:** A V8 head mount display (Virtual Research Systems, Santa Clara, Calif.) 60° diagonal  (Resolution: (640x3)x480 per eye)*  **Connected to:** Silicon Graphics Octane/MXE workstation with Octane Channel Option (Silicon Graphics, Mountain View, Calif.) | | A botanical garden in Second Life (Linden Lab, San Francisco) |
| Gujjar et al. (2019) |  |  | X | **Device:** An Oculus development kit 2 HMD (Head Mounted Display) with a resolution of 960 x 1080 per eye  (100° nominal)*  **Connected to:** VR-simulator computer (Dell XPS-8700 desktop with 4th Generation Intel Core i7-4790 processor (8 M Cache, up 4.0 GHz) and ASUS NVIDIA GEFORCE GTX 750 TI OC 2GB GDDR5 graphic card ) | | The dental environment which was possible for researcher to control and individualise |
| Lahti et al. (2020) | X |  |  | **Device:** The Samsung Gear VR headset. The 360 degrees videos (resolution range, 4,096 x 2,010 to 5,120 x 2,560).  **Connected to:** The Samsung Galaxy S7 mobile phone for the MelloVR application (weights approx. 500 g).  **Sound delivery:** played with on-ear headphones by Pioneer (model SE-M521) to exclude noise. | | The MelloVR application; video of a peaceful virtual landscape, with audio features and sound supporting the experience, which lasted 1 to 3.5 minutes. |
| Niharika et al. (2018) | X |  |  | **Device:** The VR device (Google VR Box and Anti Tank Virtual Reality 3D Glasses)  (92°-98°)*  **Sound delivery:** Headphones connected to a player capable of playing MP4 audio visual files. | | A single episode of cartoon series “Doreman” |
| Nunna et al. (2019) | X |  |  | **Device:** ANTVR Phone Glass T2  **Connected to:** Lenovo (model no: PA15LF53A, ANTV Technology Co., LTD., Beijing, China) enhanced with Theatre Max technology (that can convert any multimedia content into VR). | | Cartoons |
| Sweta et al. (2019) | X |  |  | **Device:** Head-mounted immersive type of display  **Connected to:** A smartphone | | Relaxation videos |
| Tanja-Dijsktra et al. (2014) | X |  |  | **Device:** A Wuzix iWear VR920 headset  32° field (Resolution: 640 x 480)  **Connected to:** An Alienware M11X laptop (dual-core, 1.3GHz Intel processor with Nvidia GT 540M graphics card). | | VR environment, consists of a coastal path, complete with sea, beach and field areas |
| *TOTALS* | 8 | 0 | 2 |  | |  |
| **Fear of Needles** |  |  |  |  | |  |
| Aydın et al. (2019) | X |  |  | **Device:** The headset (weighted approx. 295 g) with biconvex lenses with a diameter of 38mm, a wide viewing angle, and an optical zoom button; the interocular distance can be adjusted for children. | | 3D ‘‘Aquarium VR’’ application simulated a submarine journey to discover things underlying the virtual aquarium. |
| Chad et al. (2018) | X |  |  | **Device:** VR goggles, Tepoinn 3D Headset: support for smartphones size: 3.5-6 inches; refraction adjustment range from -500 to +200^o^; focal adjustment range: 56-70mm/2.1-2.5 inches; field of angle: 100-120^o^. | | Subjects could choose; a roller coaster ride, helicopter ride or hot-air balloon ride. |
| Dumoulin et al. (2019) | X |  |  | **Device:** A PC running on Windows XP (HP xw4600 Workstation Intel Core2 Duo CPU E6850 at 3.00 GHz, 3.48 GB of RAM, and NVDIA GeForce 8800 GTX graphic card). The immersion was provided with an eMagin z800 HMD and built in motion tracker.  (360 ^o^ horizontal field)* | | An immersive game developed by the UQO Cyberpsychology Lab using Virtools 4 |
| Dunn et al. (2019) | X |  |  | VR through iPod Touch with hand free navigation | | Immersive custom games |
| Gerçeker et al. (2019) | X |  |  | **Device:** Samsung Gear Oculus Headset  **Connected to:** the Samsung Galaxy S5 Note mobile phone | | VR- Rollercoaster -aimed to be remarkable  VR-Ocean Rift -aimed relaxation |
| Gold & Mahrer (2018) | X |  |  | **Device:** Galaxy S6 mobile-based Gear VR goggles (ages 13-21 years) or the Google Pixel mobile-based Merge VR goggles (ages 10-12 years) | | VR game Bear Blast (appliedVR^TM^) |
| Jiang et al. (2020) |  |  | X | **Device:** the Samsung Gear VR headset (Samsung electronics^tm^, 2015). Immersive VR experience through motion tracking and sound experiences.  **Connected to:** The Samsung Galaxy S7 mobile phone (Samsung electronics^tm^, 2016) | | Dental Gear VR application (International Phobia Association, 2017) |
| *TOTALS* | 7 | 0 | 0 |  | |  |
| **Claustrophobia for magnetic resonance imagining (MRI)** | | | | | | |
| Garcia-Palacios et al. (2007) | X |  |  | **Device:** A Kasier SR-80 VR helmet, and a trackball (Kensington)  **Connected to:** Dell 530 workstation with dual 2 GHz Intel CPUs, 2 GB of RAM, a GeForce 6800 video card on the Windows 2000 operating system. | | Snow world virtual reality software |
| *TOTALS* | 1 | 0 | 0 |  | |  |
| **Wound Care Anxiety** |  |  |  |  | |  |
| Chan et al. (2007) | X |  |  | **Device:** A basic VR system with super-high resolution 3-D glasses as an output device that communicates with a computer and an input/control device such as a mouse was used. | | A virtual game aimed to patrol in an ice-cream factory; included shooting foxes with an ice-cream while avoiding mistakenly shoot a little girl. |
| Das et al. (2005) | X |  |  | **Device:** Head-mount display (HMD) (IOGlasses Head Mount Display with a SVGA video resolution of 800 × 600 16 million colours) with a tracking system (Intersense IS300 6 degree of freedom Inertia Cube with a USB-Serial converter, required for Inertia Cube).  **Connected to:** A laptop (Dell Inspiration 5100, Pentium 4.2.4 Ghz CPU with a Radeon Mobility 7500 Video Card) with the game software. | | The game 'Quake' involved a visual simulation giving the children a feel of being on a track, using a pointer to aim and shoot monsters. |
| Faber et al. (2013) | X |  |  | **Device:** Cybermind Hi-Res900_ST_^TM^-3D Head Mounted Display with a field view of (Low-Tech) 31.2^O^ diagonal degree was used with an integrated audio system and controlled by a FasTrak control box. | | IVR-software SnowWorld version 2.1 (2003) |
| Hoffman et al. (2019) | X |  |  | **Device:** MX90 VR googles, from NVIS.com, with 90 degrees field of view diagonal, per eye, and 1,280 x 1,024 pixels resolution per eye. | | SnowWorld (mouse-tracking instead of head tracking) |
| Khadra et al. (2018) | X |  |  | **Device:** A projector-based VR dome environment consist of a 150^o^ wide curved screen installed at one end of the hydrotherapy tank. Operated by a projector (PANASONIC PT-RZ47, resolution: 1920x1080).  **Connected to:** A computer (Lenovo Y50-70, 2013, Version 1.0.0.52, Windows 8.1, Processor: Intel Core^TM^ i7-4720HQ). | | The video game Bubbles is a pseudo-3D game |
| Khadra et al. (2020) | X |  |  | **Device:** A projector-based VR dome environment (developed by Cobra Simulation®) consist of a 150^o^ wide curved screen installed at one end of the hydrotherapy tank. Operated by a projector (PANASONIC PT-RZ47, resolution: 1920x1080).  **Connected to:** A computer (Lenovo Y50-70, 2013, Version 1.0.0.52, Windows 8.1, Processor: Intel Core^TM^ i7-4720HQ). | | Bubbles (an interactive pseudo-3D projector dome VR videogame) |
| Konstantatos et al. (2009) |  | X |  | **Device:** VR goggles  **Connected to:** A DVD player | | Virtual Medicine |
| Maani et al. (2011) | X |  |  | **Device:** Rockwell Collins SR-80A VR goggles (Rockwell Collins, Cedar Rapids, IA).  **Connected to:** Voodoo Envy Laptop with NVDIA GForce Go 7900 GTX (512 MB) video card; Intel Core 2 Duo (T7400) CPU at 2.16 GHz, 2GB RAM at 994 MHz (HP, Palo Alto, CA). | | SnowWorld |
| McSherry et al. (2018) | X |  |  | **Device:** Virtual reality goggles (NVISINC MX 90, NVISINC, Reston, VA).  **Connected to:** A laptop computer with video card with the video program (Lenovo T 510 ThinkPad with Intel Core i7 processor and Intel HD Graphics, Leveno, Morrisville, NC).  **Sound delivered:** Earphones (Logitech Wireless Gaming Headset G930, Logitech, Freemont, CA) | | SnowWorld |
| Mott et al. (2008) | X |  |  | **Device:** The Augmented Virtual devices consists of a 7 in. LCD screen measuring 300 mm x 200 mm x 500 mm and weighting 1000g. Capable of 600 x 800 resolution and contained in a unique housing.  **Connected to:** The device requires to be connection to an Intel Pentium Trade Mark 4 computer . | | Hospital Harry; a 3-dimensional character. Manipulating the figure within the camera unit, a child can visualise the charter from multiple angles. |
| Van Twillert et al. (2007) | X |  |  | **Device:** A Cybermind Hi-Res900 3D Head Mounted Display was used with an integrated audio system and controlled by a FasTrak control-box.  (31.5° field per eye)* | | SnowWorld 2.1 |
| *TOTALS* | 10 | 1 | 0 |  | |  |

^Notes. *This information was not included in the paper.^

Appendix D

Risk of bias (RoB) assessment for randomised studies

**Risk of bias (RoB) assessment for randomised studies**

RoB has been assessed by one experienced reviewer using Cochrane RoB assessment tools. The overall quality of included studies was low. In summary, RoB assessment for the randomised controlled trials illustrated that the most common low risk of bias was with respect to selection bias (random sequence generation and allocation concealment), attrition bias, reporting bias, and performance and detection bias whilst high risk of bias was more common in blinding, ‘other’ bias and selection bias (*see* Figure 1).


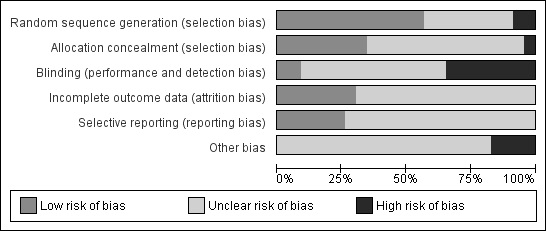


*Figure 1.* Risk of bias graph between study bias

**Selection Bias.** Most studies reported low or unknown risk for random sequence generation and allocation concealment. Studies with low risk indicated usage of randomization software or website, random numbers table or sequentially numbered opaque sealed envelopes. Only three studies reported high risk for selection bias (e.g., subjects were allocated one at a time according to their order of admission, *see* Figure 2).

**Performance and detection bias (blinding).** While unclear risk is the most common, it is followed by high risk. Only two studies was evaluated as low risk, which states blinding between deliverer and intervention, and subjects (parent and child). However, some studies stated that blinding may be challenging, especially between intervention and deliverer due to the nature of VR interventions.

**Attrition bias (incomplete outcome data).** Most studies were evaluated as unclear risk due to lack of information on how the incomplete data was handled. The remainder were rated as low risk with missingness being less than 20% of data or intention-to-treat analysis was used. No studies were rated as high risk.

**Selective reporting (reporting bias).** Most studies were evaluated as unclear risk whilst only six studies rated as low risk due to having a study protocol and reporting all their study outcomes. None of included studies were evaluated as high risk.

**Other potential sources of bias.** Nearly all studies were evaluated as unclear risk. However, only four studies were high risk, which mentioned the possibility of having a carryover bias, developing anticipated fear prior to study, and using hypnosis for only one session that may increase distress in some subjects. None of the studies were low risk.

**RoB assessment for non-randomised studies**

RoB has been evaluated by same reviewer using ROBINS-I tool. Only five studies included in this evaluation and the overall quality of these studies were moderate. Studies mostly rated as low risk of bias in the following biases; due to confounding, in selection of participants into the study, in classification of interventions, due to deviations from intended interventions, due to missing data, in measurement of outcomes, in selection of the reported result. Moderate risk was found with respect to bias in measurement outcomes, bias due to confounding, bias in selection of participants into the study.


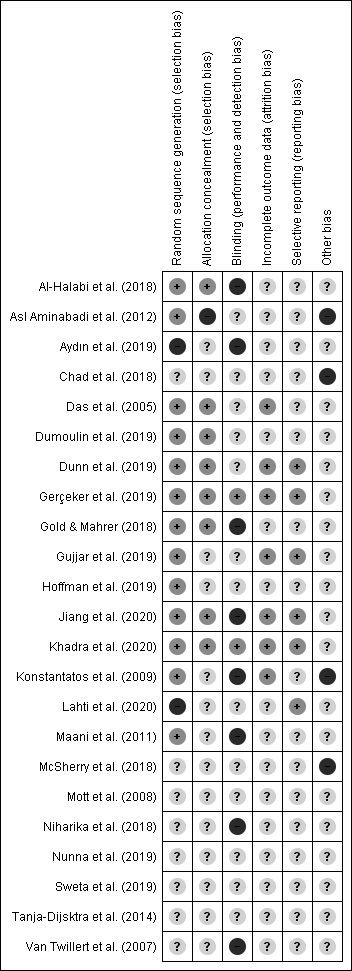


*Figure 2.* Risk of bias summary of within-study bias

Appendix E

Forest plots of effect sizes and 95% CIs for outcome measures


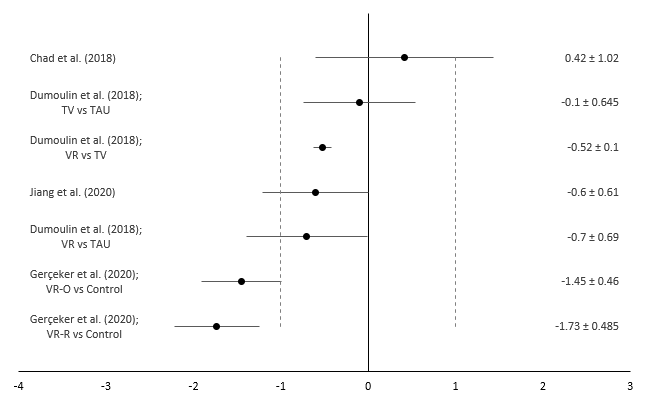


*Figure 3.* Forest plot of effect sizes and 95% CIs for fear


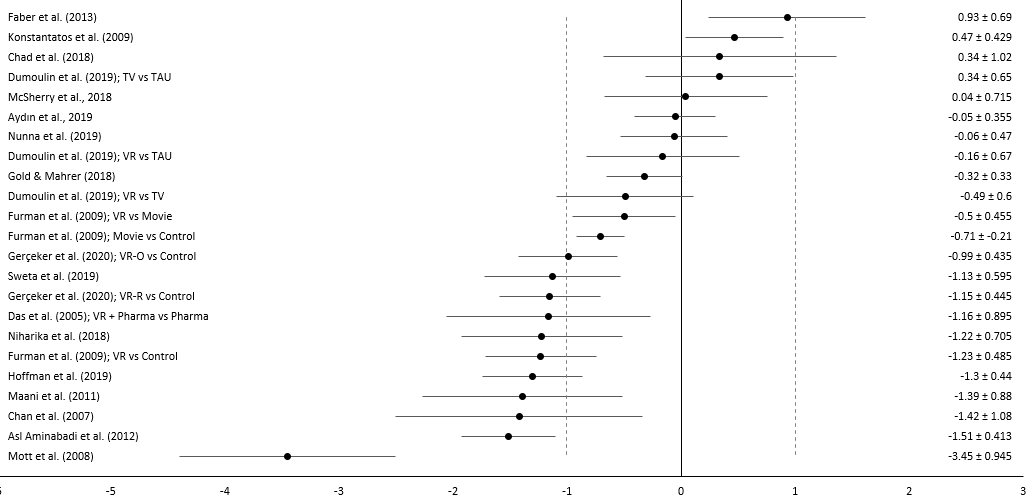


*Figure 4.* Forest plot of effect sizes and 95% CIs for pain


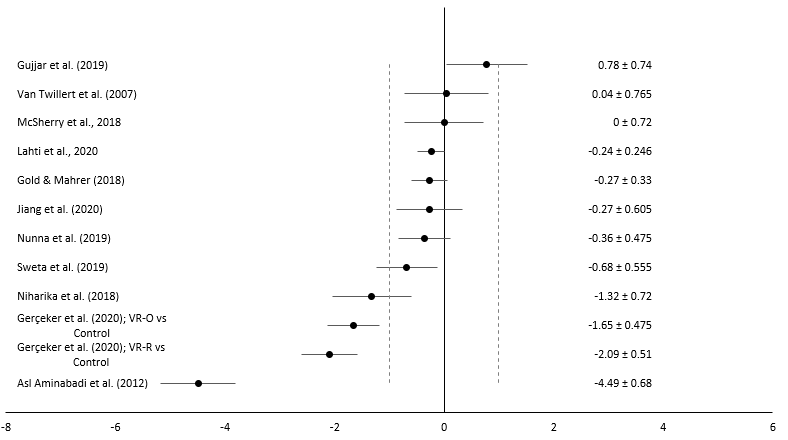


*Figure 5.* Forest plot of effect sizes and 95% CIs for anxiety
